# IN MEMORIAM-Stephen Hugh Koslow, Ph.D

**DOI:** 10.1093/ijnp/pyab025

**Published:** 2021-07-01

**Authors:** Alan Frazer, Charles B Nemeroff

**Affiliations:** 1 Department of Pharmacology, University of Texas Health Science Center, San Antonio, Texas, USA; 2 Department of Psychiatry and Behavioral Sciences, University of Texas at Austin Dell Medical School, Austin, Texas, USA


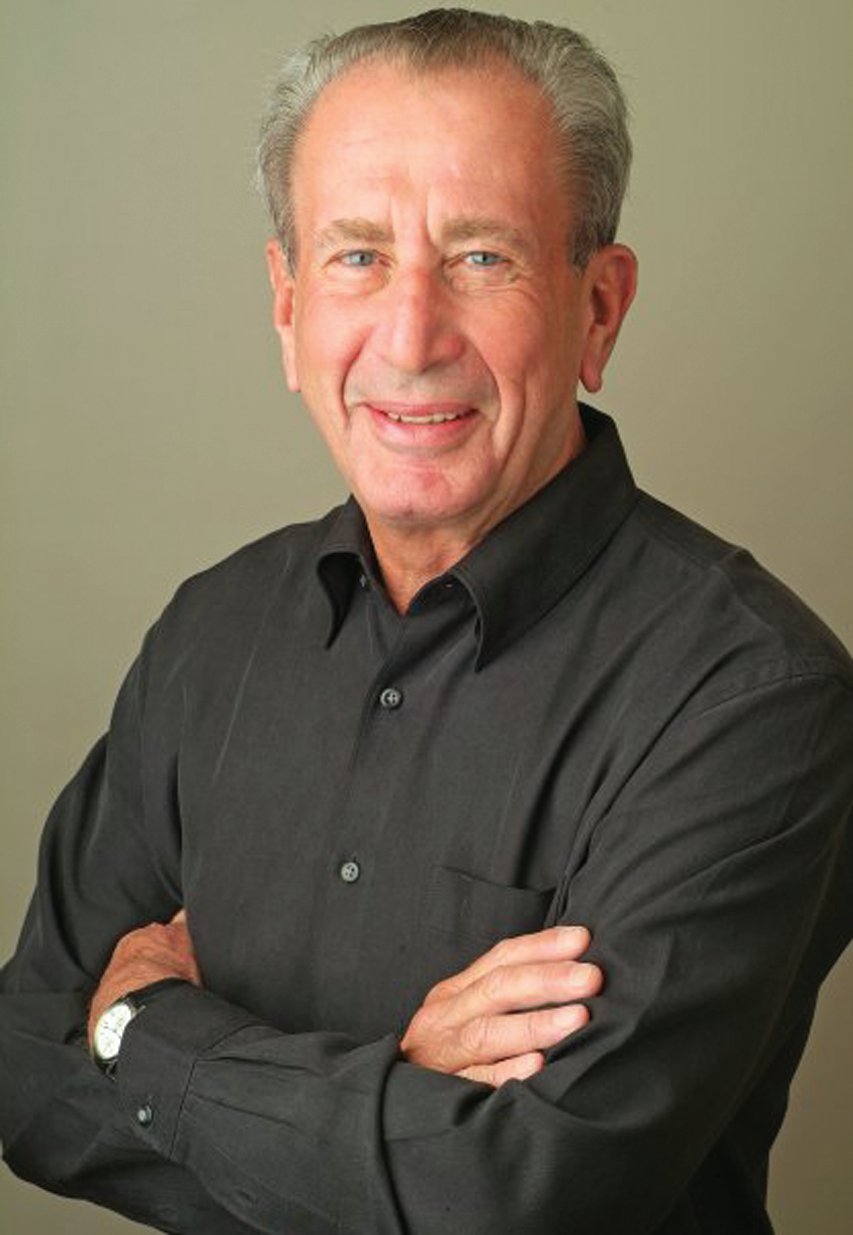


Dr. Stephen H. Koslow, a major force in the development of the neuroscience program at NIMH, died unexpectedly on April 23, 2021 while rehabilitating from a hip replacement, necessitated by a fall in early April.

Steve, a member of both the CINP and ACNP, received his Ph.D. in pharmacology from the University of Chicago and then did postdoctoral research at the Karolinska Institute in Sweden. Upon his return to the USA, he joined Erminio Costa’s Laboratory of Preclinical Pharmacology in the intramural program at NIMH and within a few years became Chief of their Unit of Neurobiological Applications of Mass Spectometry. As a true Renaissance man, Steve’s interests extended beyond the laboratory and he became very interested in the federal, and world-wide, support of biomedical research. Consequently, he moved out of the lab and became the Chief of the Biological Research Section of the Clinical Research Branch within the Division of Extramural Research programs at NIMH. Most significantly, in 1980, he became the Founding Branch Chief of the Neuroscience Research Branch in the Division of Extramural Research at NIMH. In this capacity, he tirelessly promoted the importance of both basic and clinical research into psychiatric disorders and their treatment. And he was very successful at this such that their grant portfolio increased very, very substantially during Steve’s tenure. Steve subsequently became the Director of the Division of Basic and Clinical Neuroscience and directed and managed a $250 million grant portfolio. Always looking for new fields to develop, in 1999 Steve became the Founding Director of the Office of Neuroinformatics and used this position to advance studies of computational models of neural processes, the development of tools for modeling and analyzing neuroscience data and the development of platforms through which neuroscience data could be shared. The impact this has had on our field is obvious. As was often the case, Steve was not only ahead of the curve but he threw out the first pitch. All-together, Steve served at NIMH for 24 years. He remained active after leaving NIMH, having positions at the Allen Institute for Brain Research, the American Foundation for Suicide Prevention, and the Brain Research Corporation (based in Australia).

In the early 70’s, Steve together with Marty Katz, helped to develop a six-center collaborative research program of the Psychobiology of Depression- Biological Studies. It was as a member of that long-running research group that one of us (AF) first interacted with Steve and got to know him. That professional beginning developed into an everlasting personal relationship with Steve and his family. Our children became friends during the annual Winter Conference on Brain Research meetings when we all skied together. Steve was dedicated to his family and was a champion for his children, Karin and Jamie, and that has now extended to his four grandchildren. He was very involved in their lives despite his very busy professional life which caused him to travel extensively. But Steve loved to travel, in part to renew acquaintances with his many colleagues/friends around the world but also because of his love for experiencing new places and cultures. Good food and the arts were very much a part of Steve’s life. He was trustworthy and a faithful friend. We will miss his wry sense of humor, inquisitive mind and sense of adventure.

Steve is survived by his children and grandchildren and his former spouse, Diane Koslow. In remission from stage 4 lung cancer, Steve and his partner, Nancy Levin, lived a busy and joyful life in Florida.

